# Supplementary material for: Analysis of the heat transfer fluctuations in the Rayleigh–Bénard convection of concentrated emulsions with finite-size droplets
Source: Soft Matter. 2023 Aug 28;19(37):7192–201. doi: 10.1039/d3sm00716b (PMC10523216; doi:10.1039/d3sm00716b)
Supplement: SM-019-D3SM00716B-s004 [file SM-019-D3SM00716B-s004.pdf]

Electronic Supplementary Material (ESI) for Soft Matter.
This journal is © The Royal Society of Chemistry 2023

SUPPLEMENTARY MATERIAL FOR: Analysis of the heat transfer fluctuations in the Rayleigh-Bénard convection of concentrated emulsions with finite-size droplets

Francesca Pelusi,[1,*] Stefano Ascione,[2,3] Mauro Sbragaglia,[4] and Massimo Bernaschi[1]

[1]Istituto per le Applicazioni del Calcolo, CNR - Via dei Taurini 19, 00185 Rome, Italy
[2]Department of Physics, Tor Vergata University of Rome - Via della Ricerca Scientifica 1, 00133 Rome, Italy
[3]Current affiliation: Department of Geosciences, Université de Rennes, 263 avenue Général Leclerc, 35000 Rennes, France
[4]Department of Physics & INFN, Tor Vergata University of Rome, Via della Ricerca Scientifica 1, 00133 Rome, Italy

I. THERMAL MULTI-COMPONENT LATTICE BOLTZMANN MODEL (LBM)

With the aim of simulating the dynamics of concentrated emulsions under thermal Rayleigh-Bénard convection, in this work we employ the open source code TLBfind [1]. TLBfind is based on the lattice Boltzmann method (LBM) [2, 3] for non-ideal multi-component mixtures, further supplemented with the dynamics of a temperature field coupled with that of the fluid via buoyancy forces. The same methodology has been employed in Ref. [4]. The method is briefly reviewed here.

A. Multi-component LBM

The lattice Boltzmann method is rooted in kinetic theory and it is based on the dynamical equations for the probability distribution function $f_{\sigma,i}(\mathbf{x},t)$ of finding a fluid particle of component $\sigma = 1, 2$ at the discrete time t in the lattice position $\mathbf{x}$. The fluid particles can propagate on the lattice via a discrete set of kinetic velocities $\boldsymbol{c}_i$. In our work, we employ a D2Q9 LBM scheme [2, 3] where $\boldsymbol{c}_i$ represent a set of 9 discrete velocities ($i = 0, \ldots, 8$) living on a two-dimensional lattice ($\mathbf{x} = (x, y)$). Starting form the probability distribution function, macroscopic fields, such as density and momentum, can be constructed by performing a coarse-graining process in the kinetic space as

$$\rho_\sigma(x,y,t) = \sum_i f_{\sigma,i}(x,y,t) \ , \tag{1}$$

$$\rho \boldsymbol{u}(x,y,t) = \sum_{\sigma,i} \boldsymbol{c}_i f_{\sigma,i}(x,y,t) \ , \tag{2}$$

where $\rho = \sum_\sigma \rho_\sigma$ is the global density. The dynamical evolution of $f_{\sigma,i}(x,y,t)$ is governed by the lattice Boltzmann equation

$$f_{\sigma,i}(x + c_{i,x}\Delta t, y + c_{i,y}\Delta t, t + \Delta t) - f_{\sigma,i}(x,y,t) = -\frac{\Delta t}{\tau}\left(f_{\sigma,i} - f_{\sigma,i}^{(eq)}\right)(x,y,t) + F_{\sigma,i}(x,y,t)\Delta t \ , \tag{3}$$

where Δt is the discrete simulation time step, the l.h.s. describes the streaming step while the collisional operator in the BGK approximation [5] is implemented as the first term of the r.h.s. . In Eq. (3) τ indicates the relaxation time towards the local equilibrium $f_{\sigma,i}^{(eq)}$ defined as

$$f_{\sigma,i}^{(eq)} = w_i \rho_\sigma \left[1 + \frac{u_k c_{i,k}}{c_s^2} + \frac{u_k u_p (c_{i,k} c_{i,p} - c_s^2 \delta_{kp})}{2c_s^4}\right], \tag{4}$$

where w_i are LBM weights [1], $c_s = \Delta x/(\sqrt{3}\Delta t)$ is the velocity of sound and Δx is the lattice spacing. The source term $F_{\sigma,i}(x,y,t)$ is given by the sum of two main contributions: (i) interaction forces $\boldsymbol{F}_\sigma^{\text{int}}(x,y,t)$, and ($ii$) external volume forces $\boldsymbol{F}_\sigma^{\text{ext}}(x,y,t)$. The first contribution creates the phase segregation [1, 6], thus promoting the formation

* f.pelusi@iac.cnr.it

[1] The weights w_i in the employed D2Q9 model are $w_i = 4/9$ for $i = 0$, $w_i = 1/9$ for $i = 1 \ldots 4$, $w_i = 1/36$ for $i = 5 \ldots 8$.

of stable interfaces separating the two components, and it avoids droplet coalescence by mimicking the presence of a mechanical positive disjoining pressure [1, 7, 8] [2]. In the present work, the second contribution $\boldsymbol{F}_{\sigma}^{\text{ext}}(x,y,t)$ is only given by a buoyancy term which turns on when the dynamics of the two fluids is coupled with that of the temperature, as described in the next paragraph. Thus, the total force acting on a single fluid component σ and appearing in Eq. (3) can be written as

$$\mathbf{F}_{\sigma}(x,y,t) = \boldsymbol{F}_{\sigma}^{\text{int}}(x,y,t) + \boldsymbol{F}_{\sigma}^{\text{ext}}(x,y,t). \tag{5}$$

Notice that the employed forcing scheme [6] modifies the hydrodynamical momentum of the mixture as

$$\rho \boldsymbol{u}^{(\text{H})} = \rho \boldsymbol{u} + \frac{\boldsymbol{F}^{\text{int}}\Delta t}{2} + \frac{\boldsymbol{F}^{\text{ext}}\Delta t}{2}, \tag{6}$$

where $\boldsymbol{F}^{\text{int}} = \sum_{\sigma} \boldsymbol{F}_{\sigma}^{\text{int}}$ and $\boldsymbol{F}^{\text{ext}} = \sum_{\sigma} \boldsymbol{F}_{\sigma}^{\text{ext}}$.
The long-wavelength limit of Eq. (3) approximates the Navier-Stokes equations

$$\rho\left(\partial_t + u_k^{(\text{H})}\partial_k\right)u_i^{(\text{H})} = -\partial_j P_{ij} + \eta_0 \partial_j\left(\partial_i u_j^{(\text{H})} + \partial_j u_i^{(\text{H})}\right) + \mathcal{F}_i \qquad i = \text{x,y}, \tag{7}$$

where the bulk viscosity η_0 can be directly computed in LBMs via the relaxation time τ as [2, 3]:

$$\eta_0 = \rho c_s^2 \left(\tau - \frac{\Delta t}{2}\right). \tag{8}$$

In the Navier-Stokes equations (7), $\mathcal{F}_i$ represents the volume force density and P_{ij} is the non-ideal pressure tensor, which is non-diagonal due to the contribution of interaction forces [9, 10]. Detailed calculations of P_{ij} in terms of the interaction forces are provided in Ref. [11].
Concerning the boundary conditions, in this work we impose no-slip boundary conditions at the walls ($y = \pm H/2$), which we achieve with the bounce-back rules [2, 3], and periodic boundary conditions in the x-direction.

B. Thermal LBM

In order to couple the two fluid component dynamics with that of the temperature field, a buoyancy term is introduced in Eq. (5) as external volume force in the Boussinesq's form

$$\boldsymbol{F}_{\sigma}^{\text{ext}}(x,y,t) = \rho_{\sigma}(x,y,t)\,\alpha\,g\,T(x,y,t)\,\boldsymbol{e}_y, \tag{9}$$

where $T(x,y,t)$ is the temperature field, α is the thermal expansion coefficient, g is the gravity acceleration and $\boldsymbol{e}_y$ the unit vector in the wall-to-wall direction. By following the popular strategy used in the LBM frameworks, the evolution of the temperature field

$$T(x,y,t) = \sum_i g_i(x,y,t) \tag{10}$$

can be described as done for fluid components, i.e., via the introduction of an additional probability distribution function $g_i(x,y,t)$ [12], whose dynamics reads:

$$g_i(x + c_{i,x}\Delta t, y + c_{i,y}\Delta t, t + \Delta t) - g_i(x,y,t) = -\frac{\Delta t}{\tau_g}\left(g_i - g_i^{(eq)}\right)(x,y,t), \tag{11}$$

where the local equilibrium $g_i^{(eq)}$ takes the form

$$g_i^{(eq)} = w_i T\left[1 + \frac{u_k^{(\text{H})}c_{i,k}}{c_s^2} + \frac{u_k^{(\text{H})}u_p^{(\text{H})}(c_{i,k}c_{i,p} - c_s^2\delta_{kp})}{2c_s^4}\right]. \tag{12}$$

The long-wavelength limit of (11) approximates the advection-diffusion equation for the temperature field

$$\partial_t T + u_k^{(\text{H})}\partial_k T = \kappa \partial_{kk} T, \tag{13}$$

---

[2] In this work, the values of parameters involved in the interaction forces $\boldsymbol{F}_{\sigma}^{\text{int}}(x,y,t)$ are the same as in Ref. [4].

where κ is the thermal diffusivity and it is the counterpart of the bulk viscosity in the context of the temperature dynamics since it is proportional to the thermal relaxation time τ_g as

$$\kappa = c_s^2 \left(\tau_g - \frac{\Delta t}{2} \right) . \tag{14}$$

The advection-diffusion equation (13) is two-way coupled with the Navier-Stokes equations (7) via (i) the fluid velocity field (6) entering the advection term in Eq. (13) and (ii) a buoyancy force entering in the Navier-Stokes equations as $\mathcal{F}_i = \rho\alpha g T \delta_{i,y}$, leading to the Navier-Stokes-Boussinesq equations [2, 3].

II. RHEOLOGY

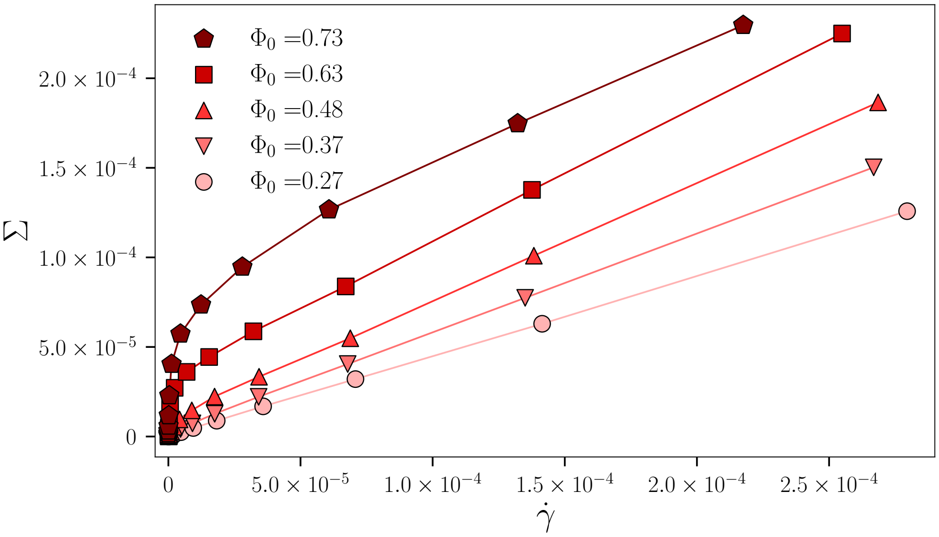

FIG. 1. Rheological characterization of emulsion systems explored in this work, from less concentrated ($\Phi_0 = 0.27$) to more concentrated emulsions ($\Phi_0 = 0.73$).

In order to characterize the rheological nature of the emulsion systems explored in this work, we performed a Couette experiment where constant and opposite velocities are imposed at the walls along the x- direction ($u_{x,wall}(y = \pm H/2, t) = \pm u_{wall}$). For each droplet concentration Φ_0, we measure the stress Σ at varying u_{wall}, resulting in different values of the the shear rate $\dot{\gamma} = 2u_{wall}/H$. Results are shown in Fig. 1. Flow curves highlight how different is the mechanical response of these emulsions, moving from Newtonian (less concentrated cases) to non-Newtonian (more concentrated emulsions).

III. CORRELATION BETWEEN DROPLET-SCALE HEAT TRANSFER FLUCTUATIONS AND DROPLET LOCALISATION

In the main text we discuss about the correlation between droplet localisation, expressed in terms of the y-coordinate of its center-of-mass-position (Y_i), and its corresponding heat transfer fluctuation $\mathrm{Nu}_i^{*,(drop)}$ (cfr. Fig. 4 of the main text). We observe that $\mathrm{Nu}_i^{*,(drop)}$ exhibits an intermittent behaviour and that there is an evident correlation between "bursts" in the droplet heat transfer fluctuation and the spatial approach-to/departure-from a wall. We also claim that a variation in the period of oscillation of Y_i is related to a layer change by the selected drop. In order to help the reader in catching this phenomenon, we include three videos, labelled as Phi027.mp4, Phi048.mp4, and Phi073.mp4, showing an animation of Fig. 4 of the main text.

[1] F. Pelusi, M. Lulli, M. Sbragaglia and M. Bernaschi, *Computer Physics Communications*, 2022, **273**, 108259.

[2] S. Succi, *The lattice Boltzmann Equation*, Oxford University Press, 2018.
[3] T. Krüger, H. Kusumaatmaja, A. Kuzmin, O. Shardt, G. Silva and E. M. Viggen, *Springer International Publishing*, 2017, **10**, 4–15.
[4] F. Pelusi, M. Sbragaglia, R. Benzi, A. Scagliarini, M. Bernaschi and S. Succi, *Soft Matter*, 2021, **17**, 3709–3721.
[5] P. L. Bhatnagar, E. P. Gross and M. Krook, *Physical review*, 1954, **94**, 511.
[6] X. Shan and H. Chen, *Physical Review E*, 1993, **47**, 1815.
[7] R. Benzi, M. Sbragaglia, S. Succi, M. Bernaschi and S. Chibbaro, *J. Chem. Phys.*, 2009, **131**,.
[8] M. Sbragaglia, R. Benzi, M. Bernaschi and S. Succi, *Soft Matter*, 2012, **8**, 10773–10782.
[9] X. Shan, *Physical Review E*, 2008, **77**, 066702.
[10] M. Sbragaglia and D. Belardinelli, *Physical Review E*, 2013, **88**, 013306.
[11] B. Dollet, A. Scagliarini and M. Sbragaglia, *J. Fluid Mech.*, 2015, **766**, 556–589.
[12] P. Ripesi, L. Biferale, M. Sbragaglia and A. Wirth, *Journal of fluid mechanics*, 2014, **742**, 636–663.
